# Intensity-modulated radiation therapy for elderly patients with esophageal cancer: Our experience

**DOI:** 10.17305/bjbms.2022.7835

**Published:** 2023-03-16

**Authors:** Dan Li, Xiaoxiao Liu, Yuchen Wang, Yingying Jin, Fang Li, Hongbing Ma

**Affiliations:** 1 Department of Radiation Oncology, The Second Affiliated Hospital of Xi’an Jiaotong University, Xi’an, China

**Keywords:** Esophageal cancer (EC), elderly, intensity-modulated radiotherapy (IMRT), treatment mode, prognosis

## Abstract

The aim of this study was to discuss the treatment mode of radical radiotherapy (RT) for elderly patients with esophageal cancer (EC). The clinical data of 136 elderly patients (≥60 years old) with EC who received radical intensity-modulated RT in The Second Affiliated Hospital of Xi’an Jiaotong University from January 2015 to December 2019 were retrospectively analyzed. Cox risk model was used for multivariate prognostic analysis, and Kaplan–Meier method was used to calculate progression free survival (PFS) and overall survival (OS). Cox regression analysis showed that ECOG score, basic diseases, T stage, radiation dose, radiation injury, and chemotherapy were the prognostic factors of elderly patients. The median OS of the RT group, concurrent chemoradiotherapy group, and sequential chemoradiotherapy group were 17, 41, and 10 months (*p* ═ 0.009), respectively. The 3-year OS and PFS of concurrent intravenous chemotherapy and oral chemotherapy were 50% and 42.9%, and 34.1% and 28.6% (*p* ═ 0.641, *p* ═ 0.702), respectively. The median OS of involved field irradiation and elective nodal irradiation (ENI) were 23 and 24 months (*p* ═ 0.219) and the local recurrence rate were 59.8% and 43.2% (*p* ═ 0.069), respectively, but the incidence and mortality of radiation pneumonia and esophagitis in ENI were higher. The 3-year OS and PFS of the low-dose group (≤60 Gy) and the high-dose group (>60 Gy) were 19.1% and 40.4%, and 14.9% and 29.2% (*p* ═ 0.012, *p* ═ 0.049), respectively. In conclusion, for elderly patients with inoperable EC, radical chemoradiotherapy should be considered a preferable selection. Among them, oral drugs and high-dose involved field irradiation exhibited better curative effects and safety.

## Introduction

Esophageal cancer (EC) is one of most common gastrointestinal (GI) tumors. The main pathological types are esophageal squamous cell carcinoma (ESCC) and esophageal adenocarcinoma [1]. In China, ESCC is the main type. EC is the eighth most common cancer and the sixth leading cause of cancer death globally [2]. In 2020, there were 324,000 new cases and 301,000 related deaths of EC in china, accounting for 53.70% and 55.35% of the global cases, respectively [3].

With the development of society and the aging of the population, the number of elderly patients with EC is gradually increasing. These patients show a low growth rate and a low distant metastasis rate. However, because it is not easy to pay attention to the early mild obstructive symptoms, once the symptoms are obvious, the disease is advanced. However, due to the basic diseases, decreased nutritional status, and body tolerance, a functional decline of various organs and other factors that lead to a high risk of surgery, radiotherapy (RT) is more acceptable. Although RTOG 8501 research established the status of concurrent chemoradiotherapy (CCRT) in non-surgical treatment of EC [4], whether the previous treatment mode, such as selective lymph node irradiation and concurrent chemotherapy is suitable for elderly patients, still need to be further discussed. This study retrospectively analyzes the prognostic factors of elderly patients with EC and aims to formulate an individualized strategy.

## Materials and methods

### Clinical data

The clinical data of 136 elderly patients with EC who received radical intensity-modulated RT (IMRT) in The Second Affiliated Hospital of Xi’an Jiaotong University from January 2015 to December 2019 were selected with the following criteria: (1) pathologically confirmed to be ESCC; (2) age ≥60, ECOG score ≤2, combined with basic diseases but stable condition (hypertension, diabetes, coronary heart disease, hepatitis, COPD, etc.), (3) the diet was liquid food or semi-liquid food; (4) all patients received radical IMRT; (5) no esophageal bleeding, perforation, and other signs; (6) no distant organ metastasis was found before treatment. According to the clinical staging, gastroscopy and imaging diagnosis and referring to the 7th edition of AJCC staging, 136 patients of stage I–IV, aged 60–89 years, with a median age of 72 years, were eligible for enrollment, 93 males and 43 females. Stage I, 5 cases, stage II, 67 cases, stage III, 27 cases, and stage IV, 37 cases. The tumors were located in the cervical segment in 10 cases, the upper segment in 16 cases, the middle segment in 74 cases, the lower segment in 36 cases, the tumor length ≤5 cm in 80 cases, and >5 cm in 56 cases.

### Radiotherapy method

Thermoplastic body film was used to fix the body position, CT enhanced scanning was used to simulate the positioning, and all patients were treated with IMRT, image guided radiation therapy (IGRT), and volumetric modulated arc therapy (VAMT).

Delineation of the target area in involved field irradiation (IFI) group: the primary tumor area (GTV) was determined according to the results of esophageal barium meal, CT, and gastroscope. The clinical target area (CTV) was 0.5–0.8 cm outward expansion of GTV axis, 1.5–2 cm upward and downward expansion, and the planned target area (PTV) was 0.5–1 cm outward expansion of CTV. The CTVnd was 0.5 cm outward expansion of metastatic lymph nodes (GTVnd) in all directions, and PTVnd was 0.5–0.8 cm outward expansion of CTVnd. In the elective nodal irradiation (ENI) group, the target area was delineated: CTV included the outward expansion of the axis of the primary focus by 0.5–0.8 cm, the upward and downward expansion by 1.5–2 cm, and the regional lymph node drainage area of this esophagus; the cervical and upper thoracic regional lymph nodes include: bilateral supraclavicular, paraesophageal, zone 2, zone 4, zone 5, and zone 7. The regional lymph nodes of the middle thoracic EC include paraesophageal, zone 2, zone 4, zone 5, zone 7, and zone 8. The regional lymph nodes of lower thoracic esophageal carcinoma include paraesophageal, zone 4, zone 5, zone 7, zone 8, zone 9, paracardial, left gastric, and peritoneal dry lymph drainage areas.

PTV is the uniform outward expansion of CTV by 0.5–1.0 cm. RT dose: 95%PTV and 95%PTVnd prescription dose 45–68 Gy, 1.8–2.0 Gy/f, 5 f/week. Normal organ limit: an average dose of both lungs ≤14 Gy, V20 ≤ 16%–26%, V30 ≤ 18%, V5 ≤ 60%; heart V40 ≤ 40%–50%, V30 ≤ 40%; spinal cord (cervical Dmax ≤ 38 Gy, other Dmax ≤ 45 Gy); stomach Dmax ≤ 50–54 Gy; small intestine V50 ≤ 10%, Dmax ≤ 50–52 Gy; kidney V20 ≤ 30%; liver V30 ≤ 30%.

### Chemotherapy

CCRT or sequential chemoradiotherapy (SCRT) is the therapy with two cisplatin-containing drugs. PF regimen: cisplatin 20–30 mg/m^2^ d1-3 + fluorouracil 800 mg/m^2^ d1-4, every four weeks as a course of treatment, and 2–4 courses of treatment according to the patient’s physical condition. TP scheme: paclitaxel 135–175 mg/m^2^ d1+ cisplatin 20–30 mg/m^2^ d1-3. Some of the older and weaker patients received concurrent single chemotherapy drug, such as S-1 and capecitabine.

**Figure 1.**
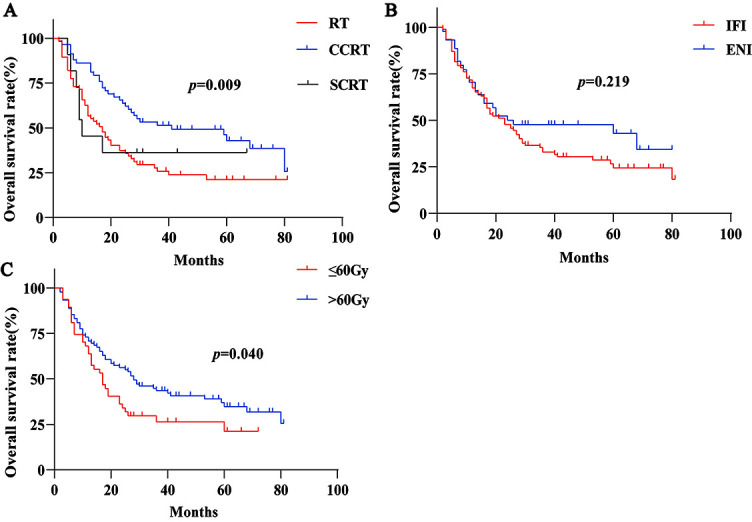
**Kaplan-Meier survival curves of different groups of esophageal cancer.** (A) Overall survival rate in patients treated with RT (*n* ═ 67) , CCRT (*n* ═ 58) and SCRT (*n* ═ 11), (B) patients treated with IFI (*n* ═ 92) and ENI (*n* ═ 44), (C) patients treated with ≤60Gy (*n* ═ 47) and >60Gy (*n* ═ 89). INI: Involved field irradiation; ENI: Elective nodal irradiation; RT: Radiotherapy; CCRT: Concurrent chemoradiotherapy; SCRT: Sequential chemoradiotherapy.

### Follow-up and efficacy evaluation

Follow-up was conducted by telephone and outpatient follow-up. The deadline for follow-up was December 31, 2021. The endpoints were overall survival (OS) and progression free survival (PFS). OS is defined as the time interval from the beginning of treatment to the death of the patient due to any cause. PFS is defined as the time interval from the end of treatment to the occurrence of tumor recurrence, progression, or death from any cause.

### Statistical analysis

SPSS 25.0 software was used to perform x2 test on count data, t-test was used for calculation data, Cox proportional hazard model was used for multivariate analysis, Kaplan–Meier method was used to calculate survival rate, and log-rank method was used to test. *P* < 0.05 was statistically significant.

## Results

### Survival analysis

The follow-up time of the whole group was 2–81 months, the median follow-up time was 59 months, the median OS was 23 months, and the median PFS was 19 months. The median OS of the RT, CCRT, and SCRT group were 17, 41, and 10 months (*p* ═ 0.009, Figure 1A), respectively. The 3-year OS and PFS of concurrent intravenous chemotherapy and oral chemotherapy were 50% and 42.9%, and 34.1% and 28.6% (*p* ═ 0.641, *p* ═ 0.702), respectively. The median survival time of IFI and ENI were 23 and 24 months (*p* ═ 0.219, Figure 1B), respectively. The 3-year OS of IFI and ENI were 31.5% and 36.4% (*p* ═ 0.575), respectively. The 3-year PFS of two groups were 23.9% and 25.0% (*p* ═ 0.890), respectively. The incidence and mortality of radiation pneumonia and esophagitis in ENI were higher. The median OS of the low dose group (≤60 Gy) and high dose group (>60 Gy) were 17 and 28 months (*p* ═ 0.040, Figure 1C), the 3-year OS of the two groups were 19.1% and 40.4% (*p* ═ 0.012), respectively. The PFS of the two groups were 14.9% and 29.2% (*p* ═ 0.049), respectively. The median OS of ≤50.4 Gy and 51–60 Gy group were 6 and 19 months, respectively (*p* ═ 0.001).

### Univariate and multivariate analysis of prognosis

Univariate analysis showed that age, ECOG score, comorbidity, stage, T stage, radiation dose, chemotherapy, and radiation injury were related to the 3-year OS rate of elderly patients with EC (Table 1). Cox regression analysis demonstrated that the independent risk factors for the 3-year OS rate were ECOG score (hazard ratio [HR] ═ 3.016, 95% confidence interval [CI] 1.860–4.891, *p* ═ 0.006), comorbidity (HR ═ 1.778, 95% CI 1.171–2.700, *p* ═ 0.007), T stage (HR ═ 1.423, 95% CI 1.124–1.801, *p* ═ 0.003), radiation dose (HR ═ 0.694, 95% CI 0.481–1.000, *p* ═ 0.050), chemotherapy (HR ═ 0.530, 95% CI 0.344–0.815, *p* ═ 0.004), and complications (HR ═ 1.679, 95% CI 1.047–2.694, *p* ═ 0.032), as demonstrated in Table 2.

### Subgroup analysis

**Figure 2.**
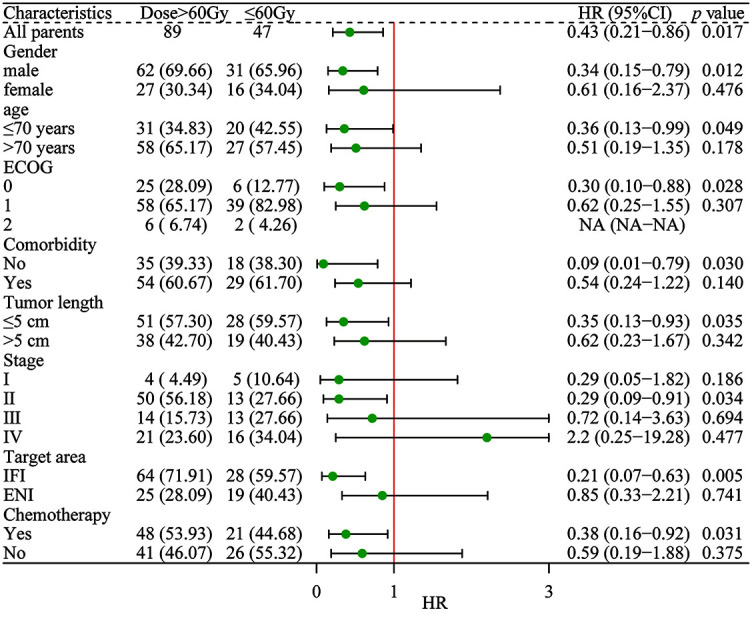
**Subgroup survival analysis in patients treated with ≤60Gy (*n* ═ 47) and >60Gy (*n* ═ 89).** ECOG: Eastern cooperative oncology group; Stage: The 7th edition of AJCC staging; INI: Involved field irradiation; ENI: Elective nodal Irradiation; RT: Radiotherapy; CCRT: Concurrent chemoradiotherapy; SCRT: Sequential chemoradiotherapy.

In the subgroup analysis of the high-dose group (>60 Gy), patients with gender, age, ECOG score, combined basic diseases, tumor length, stage, and chemotherapy showed better survival efficacy (Figure 2). The 3-year OS and PFS for patients with aged ≤70 years were 58.1% and 45.2% (*p* ═ 0.013, *p* ═ 0.016), respectively. The 3-year OS and PFS for ECOG score ═ 0 group were 76% and 60% (*p* ═ 0.000, *p* ═ 0.000), respectively. The 3-year OS and PFS for the T1-2N0M0 group 86.7% and 60% (*p* ═ 0.000, *p* ═ 0.039), respectively. The 3-year OS and PFS for patients without basic diseases were 55.6% and 40.7% (*p* ═ 0.000, *p* ═ 0.000), respectively.

### Complications

We retrospectively evaluated the acute radiation injury induced GI toxicities, radiation esophagitis, and pneumonia using the RTOG acute radiation injury classification standard and the Common Terminology Criteria for Adverse Events (CTCAE) v.5. Most patients had GI toxicities, but there were no serious adverse events. The incidence of grade 3-4 myelosuppression in the RT and CRT group was 13.4% and 46% (*p* ═ 0.000, CTCAE v.5 criteria), respectively, which did not lead to death, while the incidence of grade 3 and above radiation esophagitis and pneumonia in the IFI group and ENI group was 19.8% and 31.8% (*p* ═ 0.115, Table 3), respectively. Mortality was 27.8% and 78.6% (*p* ═ 0.011), and the incidence of esophageal perforation was higher in the ENI group.

## Discussion

According to National Comprehensive Cancer Network (NCCN) guidelines, the standard treatment for early-stage EC is both surgery and CRT, locally advanced EC is mainly treated with comprehensive treatment, including neoadjuvant chemotherapy or chemoradiotherapy, surgery, RT, and chemoradiotherapy. However, the treatment for elderly patients with EC has been controversial, standard treatment or optional treatment is selected by chronological age, physical condition, and patient preference. Some studies have shown that old age is the main factor affecting prognosis and treatment toxicity (HR ═ 4.93, 95%CI 1.03–23.64, *p* ═ 0.046) [5]. Han et al. [6] conducted a meta-analysis to compare the clinical outcomes of esophagectomy between elderly patients and non-elderly patients. They found that esophagectomy for elderly patients had a higher risk of in-hospital mortality (OR ═ 2.00, 95%CI 1.28–3.13, *p* ═ 0.002), higher incidence rates of cardiac (OR ═ 1.55, 95% CI 1.10–2.20, *p* ═ 0.01), or pulmonary complications (OR ═ 1.57,95% CI 1.11–2.22, *p* ═ 0.01), and lower survival rates (OR ═ 2.66, 95%CI 1.65–4.28, *p* < 0.001). Therefore, the study found that a cancer-specific comprehensive geriatric assessment model should be developed, which is more conducive to formulating a plan. For patients with locally advanced EC, surgery or other treatments should be selected according to the scores after RT and chemotherapy [7, 8].

**Table 1:** Univariate prognosis analysis of 3-year overall survival in elderly patients with esophageal cancer

**Characteristic**	***n***	**OS, % (n)**	**X^2^**	***P***
*Age (years)*				
≤70	51	45.1 (23)	5.316	0.021
>70	85	25.9 (22)		
*Sex*				
male	93	36.6 (34)	1.601	0.206
female	43	25.6 (11)		
*ECOG score*				
0	31	71.0 (22)	29.055	0.000
1	97	23.7 (23)		
2	8	0.0 (0)		
*Comorbidity*				
No	83	44.6 (37)	12.7	0.000
Yes	53	15.1 (8)		
*Stage*				
I	5	100 (5)	20.195	0.000
II	67	41.8 (28)		
III	27	22.2 (6)		
IV	37	16.2 (6)		
*T Stage*				
1	5	100 (5)	24.516	0.000
2	16	68.8 (11)		
3	78	29.5 (23)		
4	37	16.2 (6)		
*Tumor site*				
cervical	10	40.0 (4)	3.029	0.387
upper	16	31.3 (5)		
middle	74	37.8 (28)		
lower	36	22.2 (8)		
*Tumor length (cm)*				
≤5	80	37.5 (30)	1.708	0.191
>5	56	26.8 (15)		
*Chemotherapy*				
Yes	69	43.5 (30)	6.829	0.009
No	67	22.4 (15)		
*Target area*				
IFI	92	31.5 (29)	0.315	0.575
ENI	44	36.4 (16)		
*Radiation Dose*				
≤60Gy	47	19.1 (9)	6.303	0.012
>60Gy	89	40.4 (36)		
*Radiation injury (Grade)*				
≤2	106	38.7 (41)	6.785	0.009
>3	30	13.3 (4)		
*Myelosuppression (Grade)*				
≤2	95	33.7 (32)	0.051	0.822
≥3	41	31.7 (13)		

ECOG: Eastern Cooperative Oncology Group; Comorbidity: Hypertension, diabetes, coronary heart disease, hepatitis, COPD, etc.; Stage: The 7th edition of AJCC staging; Radiation injury: Radiation pneumonia and esophagitis (RTOG acute radiation injury classification standard); Myelosuppression: The Common Terminology Criteria for Adverse Events (CTCAE).

**Table 2:** Multivariate cox regression analysis of overall survival in elderly patients with esophageal cancer

	**HR**	**95%CI**		***p***
ECOG	3.016	1.860	4.891	0.000
Comorbidity	1.778	1.171	2.700	0.007
T Stage	1.423	1.124	1.801	0.003
Radiation Dose	0.694	0.481	1.000	0.050
Chemotherapy	0.530	0.344	0.815	0.004
Complications	1.679	1.047	2.694	0.032

ECOG: Eastern Cooperative Oncology Group; Comorbidity: hypertension, diabetes, coronary heart disease, hepatitis, COPD, etc.; Stage: The 7th edition of AJCC staging; Complications: Radiation pneumonia and esophagitis (RTOG acute radiation injury classification standard); Myelosuppression: the Common Terminology Criteria for Adverse Events (CTCAE).

In our study, the main treatments were RT alone, CCRT, and SCRT. The median OS of three groups were 17, 41, and 10 months (*p* ═ 0.009), respectively. The 2-year OS of concurrent intravenous chemotherapy and oral chemotherapy were 70.5% and 50% (*p* ═ 0.161), respectively, the 2-year PFS of the two groups were 54.5% and 35.7% (*p* ═ 0.220), respectively. The 3-year OS of two groups were 50% and 42.9% (*p* ═ 0.641), respectively, the 3-year PFS of two groups were 34.1%, and 28.6% (*p* ═ 0.702), respectively. Intravenous chemotherapy showed a better curative effect among CCRT group, but its hematotoxicity and GI toxicity were higher. The incidence of grade 3 and above myelosuppression was 56.8%, while the oral S-1 or capecitabine group was only 7.1% (*p* ═ 0.000). Some patients may not be able to tolerate CCRT because of their basic diseases and poor constitution. Therefore, SCRT were performed, but the curative effect was poor, the 3-year OS was only 18.2%. A previous meta-analysis of EC showed that there were significant differences in 2- and 3-year survival rates between CCRT and SCRT (OR ═ 2.26, 95%CI:1.79–2.86, *p* < 0.00001; OR ═ 2.37, 95%CI:1.88–3.00, *p* < 0.00001) [9], CCRT has a synergistic effect, and chemotherapy will increase the sensitivity of RT. In RTOG 8501 study, the 5-year OS rate of CCRT and RT group were 26% and 0%, which established the status of CCRT in non-surgical treatment of EC. However, cisplatin combined with fluorouracil had significant toxicity and side effects, with 62.5% of grade 3 and above adverse events [4]. Several previous studies have demonstrated that definitive CRT might be considered as both effective and safe in elderly patients with EC, exhibiting similar higher rates of clinically response compared to younger patients [10, 11]. If patients cannot tolerate doublet CT combined with RT, single-drug oral chemotherapy drugs can be considered, such as S1, Xeloda, and other fluorouracil analogs [12, 13]. Ji et al. [13] reported that the RT with S-1 group had a significantly higher complete response rate than the RT group (41.6% vs 26.8%, *p* ═ 0.007) as well as a significantly higher 2-year OS rate (53.2% vs 35.8%, HR ═ 0.63, 95% CI 0.47–0.85, *p* ═ 0.002). There were no significant differences in the incidences of grade 3 or higher toxic effects between the two groups (9.5% vs 2.7%, *p* ═ 0.01). Therefore, we should formulate individualized therapy according to the elderly patients’ ECOG score, age, basic diseases, tumor type, etc. CCRT with oral drugs can be considered as both effective and safe.

Retrospective studies in Japan have shown that the complete response rate of non-surgical treatment based on radical RT for stage I EC is more than 85% [14–17]. In the JCOG 9708 study [14], 72 patients with T1N0M0 EC received 60 Gy RT plus 2 cycles of cisplatin combined with fluorouracil concurrent chemotherapy. The 4-year OS rate and relapse-free survival rate were 80.5% (95% CI 71.3–89.7) and 68% (95% CI 57.3–78.8), respectively, similar to the surgical efficacy. Wang et al. [18] reported that the 5-year survival rate of patients with stage I-IIA EC was 56.2%–84.9%. In our study, patients with T1-2N0M0 received chemoradiotherapy, the 3-year OS and PFS were 86.7% and 60%, respectively (*p* ═ 0.000, *p* ═ 0.039), which was similar to previous studies. Therefore, CRT seems to be an alternative treatment option for stage I-II EC, because its efficacy is comparable with that of surgery, patients can expect a better quality of life.

**Table 3:** Toxicities in different groups

**Characteristic**	*n*	**Grade 3-4 myelosuppression (%)**	***p***	**Grade 3-5 radiation injury (%)**	***p***
***Treatment***					
RT	67	13.4	0.000	29.9	0.087
CRT	69	46.4		17.4	
***Target area***					
IFI	92	27.2	0.375	19.6	0.115
ENI	44	36.4		31.8	

INI: Involved field irradiation; ENI: Elective nodal irradiation; RT: Radiotherapy; CCRT: Concurrent chemoradiotherapy; Radiation injury: Radiation pneumonia and esophagitis (RTOG acute radiation injury classification standard); Myelosuppression: The Common Terminology Criteria for Adverse Events (CTCAE).

EC has the characteristics of micrometastasis and jumping metastasis and may have a multifocal origin. The law of lymph node metastasis is not clear, and it is difficult to predict. Therefore, treatment failure may be caused by distant metastasis or local recurrence [19, 20]. There has been controversy over the use of IFI or ENI in the RT range. Fewer studies reported that three field lymph node dissection in surgical treatment was effective in reducing the incidence of micrometastasis, and the survival rate and local control rate were significantly increased. Therefore, it is of great significance to appropriately increase the radiation range of RT to reduce the recurrence rate of lymph nodes [21]. Several prospective and retrospective studies reported that the efficacy of IFI was basically similar to ENI, but adverse events were significantly less than ENI. Compared with ENI, IFI has showed similar efficacy and reduced radiation-induced toxicity [22, 23]. The recurrence rate of irradiation field area with INI group was only 2%–12.5% [24, 25]. The RT target adopted by RTOG 0113 research in the United States and SCOPE 1 research in the United Kingdom was also a similar IFI technology [26, 27]. In our study, there was no significant difference between the median survival time of IFI and ENI (23 vs 24 months, *p* ═ 0.219), the 2-year and 3-year OS were 47.8% and 52.3%, and 1.5% and 36.4% (*p* ═ 0.628, *p* ═ 0.575), respectively. The local recurrence rate was 59.8% and 43.2% (*p* ═ 0.069), and the distant metastasis rate was 12.0% and 13.6% (*p* ═ 0.782), respectively. Although there was no statistical difference between the recurrence and metastasis rates of the two irradiation methods, it seems to indicate ENI reduced the local recurrence rate of patients. However, the incidence of grade 3 and above radiation pneumonia and esophagitis in 44 patients with ENI was 31.8% (*n* ═ 14), and the mortality was 78.6% (*n* ═ 11). In 92 patients with the IFI group, the toxicity was 19.5% (*n* ═ 18), and the mortality was 33.3% (*n* ═ 6). Comparatively, ENI treatment was easy to lead to serious complications and high mortality, increase the economic burden of patients and decrease the quality of life. The long-term efficacy of INI was similar to ENI and the incidence of severe radiation pneumonia and esophagitis were fewer. Therefore, IFI treatment could be considered for elderly patients.

The dose of radical RT for EC has always been controversial. The total dose of radical RT in Europe and the United States is 50–50.4 Gy, while in China and Japan, the fractional dose of 60–70 Gy is 1.8–2.0 Gy/time [4, 28]. Among 218 patients with EC in RTOG 9405 study, the high-dose radical chemoradiotherapy group (64.8 Gy) was not better than the low-dose group (50.4 Gy) in survival or local control, and even worse than the low-dose group (31% vs. 40%) in median survival and absolute 2-year survival rate. Therefore, RTOG 9405 research has laid the foundation for European and American countries to take 50–50.4 Gy as the standard RT dose for EC CCRT [29], however, the efficacy was not optimistic. The 2-year OS rates were only 36%–56%, the 3-year OS rates were 26.9%–33%. There were 11 treatment-related deaths in the high-dose group and only 2 in the low-dose group. However, 8 of the 11 patients in the high-dose group died when the dose was ≤54 Gy, and the earliest death occurred when the dose was 5.4 Gy (3 times). So many deaths were not caused by dose toxicity. The JOCG 0303 study showed that compared with the low dose (50 Gy) group, the high dose (60 Gy) group did not increase the OS rate of patients, but the study results were still controversial [30].

Xu et al. [31] compared the efficacy of 60 Gy RT dose and 50 Gy RT dose for EC. The study showed that there was no significant statistical difference between the high and low groups in terms of local recurrence, PFS, and OS, which was similar to the research results of RTOG 9405. Chang et al. [32] showed that among 2061 patients with ESCC who received IMRT-based CCRT, RT (≥60 Gy) in the high-dose group improved the OS rate (35.47% vs 26.74%, *p* < 0.0001) compared with the low-dose group. Our results showed that the efficacy of the high-dose group was significantly better than that of the low-dose group. The median OS of ≤60 Gy and >60 Gy were 17 and 28 months (*p* ═ 0.040), respectively. The 2-year OS and PFS were 36.2% and 56.2%, and 27.7% and 46.1% (*p* ═ 0.026, *p* ═ 0.037), and the 3-year OS and PFS were 19.1% and 40.4%, and 14.9% and 29.2% (*p* ═ 0.012, *p* ═ 0.049), respectively. The median OS of ≤50.4 Gy and 51–60 Gy groups was 6 and 19 months, respectively (*p* ═ 0.001). Subgroup analysis showed that the survival effect of high-dose group was better in gender, age, ECOG score, combined basic diseases, tumor length, stage, and chemotherapy.

Chen et al. [33] published the research results on the comparison of the curative effects of different chemotherapy regimens in radical CCRT for EC. The results showed that RT (61.2 Gy/34f) combined with two different chemotherapy regimens exhibited long-term clinical benefits. Considering that the efficacy of previous high-dose RT was inferior to the low-dose RT, the main reasons for the difference in survival were as follows: (1) European patients included in RTOG 9405 are mainly esophageal adenocarcinoma, while most Asian patients with EC cancer are squamous cell carcinoma; (2) 2D-RT RT technology was used in RTOG 9405 study. At present, 3DCRT and IMRT are widely used. Previous studies have shown that 3D IMRT/DCRT technology can improve the local control rate and OS rate of EC patients compared with 2D-RT technology. With the increase of the dose in the PTV of 3D IMRT group, the radiation dose in spinal cord, lung, and heart was significantly reduced, and the incidence of radiation esophagitis and pneumonia was significantly reduced [34–35]; (3) Different chemotherapy drugs have different toxic and side effects, which are also the main factors affecting the prognosis. Considering that 3D RT techniques, such as IMRT and 3DCRT, have optimized the dose uniformity of tumor target area and protected the normal tissues compared with 2D-RT technology, the toxicity of RT was significantly reduced. It is controversial whether the results of RTOG 9405 clinical study with 2D-RT technology are applicable to the era of 3D IMRT. Therefore, a larger multicenter prospective trial need to validate our findings and confirm radiation dose of ESCC in China.

## Conclusion

In conclusion, ECOG score, basic disease, T stage, radiation dose, chemotherapy, and radiation injury were the prognostic factors of elderly patients with EC. Age is not a restrictive condition for treatment options, and we should take into account survival benefits and patient preferences. For elderly patients with locally advanced or inoperable EC, radical CRT should be considered a preferable selection. Among them, oral chemotherapy drugs and high-dose IFI exhibited better curative effects and safety. For elderly patients with low ECOG score, young age, early stage, and no basic disease, high-dose irradiation showed better clinical benefits.
